# Supplementary material for: Single nucleotide substitutions effectively block Cas9 and allow for scarless genome editing in Caenorhabditis elegans
Source: Genetics. 2021 Nov 15;220(1):iyab199. doi: 10.1093/genetics/iyab199 (PMC8733430; doi:10.1093/genetics/iyab199)
Supplement: iyab199_Supplementary_Data [file iyab199_supplementary_data.pdf]

A

```
                               Cas9 guide sequence [P8]
mir-1 genomic:     GATATGGAATGTAAAGAAGTATGTAGAACGGGGTGGTAGT
Repair template:   GATATGGAATGTAAAGAAGTATTTAGAACGGGGTGGTAGT
                       *                          PAM
```

```
     -A      A        UA              -A      A        UUA
  UGG  AUGUAA GAAGUAUG             UGG  AUGUAA GAAGUAU
  ACC  UACAUU CUUCAUAC             ACC  UACAUU CUUCAUA
AU   CG      C                   AU   CG      C        C
```

wild-type miR-1 duplex — **UY477: *mir-1(zen208)***

B

```
                                  Cas9 guide sequence [P2]
mir-84 genomic:    TACTATTCATCATACGTCTGCCTGTGGCATCTGAGGTAGT
Repair template:   TACTATTCATCATACGTCTGCCTGTGGCATCCGAGGTAGT
                                                  *  PAM
```

```
       G    A  U         AGA              G    A  U         AGA
  UGAG UAGU UG AAUAUUGU                CGAG UAGU UG AAUAUUGU
  GCUC AUCA AC UUGUAACA                GCUC AUCA AC UUGUAACA
CG     A    -  U         C           CG     A    -  U         C
```

wild-type miR-84 duplex — **UY459: *mir-84(zen194)***

Figure S1: Examples of CRISPR-generated alleles using single nucleotide substitutions in the guide-binding region. (A) Partial alignment of the ssODN repair template used to mutate *mir-1* compared to the wild-type *mir-1* genomic sequence (Top). The resulting strain (UY477) carries a single nucleotide change of the 8th nucleotide from the 3' end of the guide sequence. (Bottom) Comparison of the wild-type *mir-1* duplex and the UY477 mutated *mir-1* duplex. (B) Partial alignment of the ssODN repair template used to mutate *mir-84* compared to the wild-type *mir-84* genomic sequence (Top). The resulting strain (UY459) contains a single nucleotide change at the 2nd nucleotide from the 3' end of the guide sequence. (Bottom) Comparison of the wild-type *mir-84* duplex and the UY459 mutated *mir-84* duplex. (A-B) Changes to the wild-type sequence are indicated by red text. The PAM sequence is double underlined, and the guide sequence is highlighted by a gray box. Asterisk indicates the 5' nucleotide of the mature microRNA guide strand. Duplexes were derived from www.mirbase.org.

Table S1. List of Strains Used in This Study

Strain	Genotype	Information
FX30240	*tmc24 [F23D12.4(tmIs1240)] X*	Dejima et al., 2018
N2	wild-type	From CGC
UY352	*tra-2(zen142) II*	PAM + *Rsa*I
UY356	*tra-2(zen145) II*	*Rsa*I only, no blocking
UY362	*tra-2(zen151) II*	P2 + *Rsa*I
UY364	*tra-2(zen153) II*	P11 + *Rsa*I
UY370	*tra-2(zen157) II*	P20 + *Rsa*I
UY386	*let-7(zen162)/tmc24 X*	PAM
UY389	*let-7(zen165)/tmc24 X*	No blocking
UY392	*let-7(zen168)/tmc24 X*	*let-7(n2853*-equivalent)
UY440	*ndf51 V; let-7(zen171) mir-84(n4037)/tmc24 X*	*let-7* family mutant
UY459	*mir-84(zen194) X*	P2, see Figure S1
UY477	*mir-1(zen208) I*	P8, see Figure S1
VT1066	*ndf51 V; mir-84(n4037) X*	Abbott et al., 2005

Table S2. List of Oligonucleotides Used in This Study

Oligonucleotide	Sequence (5'-3')
tra-2 crRNA	AUUUUACUAACAGAUAAUAA
let-7 crRNA	UGUGGAUCCGGUGAGGUAGU
dpy-10 crRNA	UUCUGCUGUCUUGAUUGACG
tra-2 PAM ssODN	CCTACAAATTATTTTGAGTAATATTTTTATTTACGATATTATTT TACTAACAGATAATAATCGTACAATGAAATTGAAATACAATA AACTTCTCGTTTCGGTGG
tra-2 No-Block ssODN	CCTACAAATTATTTTGAGTAATATTTTTATTTACGATATTATTT TACTAACAGATAATAATGGTACAATGAAATTGAAATACAATA AACTTCTCGTTTCGGTGG
tra-2 P2 ssODN	CCTACAAATTATTTTGAGTAATATTTTTATTTACGATATTATTT TACTAACAGATAATTATGGTACAATGAAATTGAAATACAATA AACTTCTCGTTTCGGTGG
tra-2 P2+PAM ssODN	CCTACAAATTATTTTGAGTAATATTTTTATTTACGATATTATTT TACTAACAGATAATTATCGTACAATGAAATTGAAATACAATA AACTTCTCGTTTCGGTGG
tra-2 P5 ssODN	CCTACAAATTATTTTGAGTAATATTTTTATTTACGATATTATTT TACTAACAGATTATAATGGTACAATGAAATTGAAATACAATA AACTTCTCGTTTCGGTGG

tra-2 P8 ssODN	CCTACAAATTATTTTGAGTAATATTTTTATTTACGATATTATTT<br>TACTAACACATAATAATGGTACAATGAAATTGAAATACAATA<br>AACTTCTCGTTTCGGTGG
tra-2 P11 ssODN	CCTACAAATTATTTTGAGTAATATTTTTATTTACGATATTATTT<br>TACTATCAGATAATAATGGTACAATGAAATTGAAATACAATA<br>AACTTCTCGTTTCGGTGG
tra-2 P11+PAM ssODN	CCTACAAATTATTTTGAGTAATATTTTTATTTACGATATTATTT<br>TACTATCAGATAATAATCGTACAATGAAATTGAAATACAATA<br>AACTTCTCGTTTCGGTGG
tra-2 P14 ssODN	CCTACAAATTATTTTGAGTAATATTTTTATTTACGATATTATTT<br>TAGTAACAGATAATAATGGTACAATGAAATTGAAATACAATA<br>AACTTCTCGTTTCGGTGG
tra-2 P17 ssODN	CCTACAAATTATTTTGAGTAATATTTTTATTTACGATATTATT<br>ATACTAACAGATAATAATGGTACAATGAAATTGAAATACAAT<br>AAACTTCTCGTTTCGGTGG
tra-2 P20 ssODN	CCTACAAATTATTTTGAGTAATATTTTTATTTACGATATTTTTT<br>TACTAACAGATAATAATGGTACAATGAAATTGAAATACAATA<br>AACTTCTCGTTTCGGTGG
tra-2 P20+PAM ssODN	CCTACAAATTATTTTGAGTAATATTTTTATTTACGATATTTTTT<br>TACTAACAGATAATAATCGTACAATGAAATTGAAATACAATA<br>AACTTCTCGTTTCGGTGG
let-7 PAM ssODN	GATTGGTGGACGGTCTACACTGTGGATCCGGTGAGGTAGT<br>ACGTTGTATAGTTTGGAATATTACCACCGGTGAACTATGCA

let-7 no-Block ssODN	GATTGGTGGACGGTCTACACTGTGGATCCGGTGAGGTAGT AGGTTCTATAGTTTGGAATATTACCACCGGTGAACTATGCA
let-7(n2853) ssODN	GATTGGTGGACGGTCTACACTGTGGATCCGGTGAGATAGT AGGTTGTATAGTTTGGAATATTACCACCGGTGAACTATGCA
tra-2 RsaI Reversion ssODN	CCTACAAATTATTTTGAGTAATATTTTTATTTACGATATTATTT TACTAACAGATAATAATGGAACAATGAAATTGAAATACAATA AACTTCTCGTTTCGGTGG
dpy-10(cn64) ssODN	CACTTGAACTTCAATACGGCAAGATGAGAATGACTGGAAAC CGTACCGCATGCGGTGCCTATGGTAGCGGAGCTTCACATG GCTTCAGACCAACAGCCTAT

Table S3 (Related to Figure 2). Editing Rates of *tra-2* in F2 Generation Dumpy (top) and Non-dumpy (bottom) Animals.

	**Blocking Mutation**	**HDR Edited (%)**	**Indel (%)**	**Not Edited (%)**
Genotypes of F2 Dumpy	No-blocking	19 (21.8)	8 (9.2)	60 (69.0)
	PAM	42 (95.4)	1 (2.3)	1 (2.3)
	P2	40 (80.0)	1 (2.0)	9 (18.0)
	P2+PAM	45 (93.8)	0 (0.0)	3 (6.2)
	P5	50 (75.8)	1 (1.5)	15 (22.7)
	P8	22 (67.6)	4 (11.8)	7 (20.6)
	P11	28 (75.7)	3 (8.1)	6 (16.2)
	P11+PAM	31 (88.6)	2 (5.7)	2 (5.7)
	P14	39 (59.1)	8 (12.1)	19 (28.8)
	P17	23 (54.8)	5 (11.8)	14 (33.4)
	P20	28 (66.6)	4 (9.6)	10 (23.8)
	P20+PAM	34 (89.5)	0 (0.0)	4 (10.5)
Genotypes of F2 non-Dumpy	No-blocking	2 (2.3)	6 (6.9)	79 (90.8)
	PAM	3 (6.8)	6 (13.6)	35 (79.6)
	P2	7 (14.0)	9 (18.0)	34 (68.0)
	P2+PAM	5 (10.4)	5 (10.4)	38 (79.2)
	P5	4 (6.1)	5 (7.6)	57 (86.3)
	P8	2 (5.9)	2 (5.9)	30 (86.4)

P11	5 (13.5)	1 (2.7)	31 (83.8)
P11+PAM	5 (14.3)	4 (11.4)	26 (74.3)
P14	1 (1.5%)	7 (10.6)	58 (87.9)
P17	3 (7.2%)	7 (16.6)	32 (76.2)
P20	6 (14.3)	6 (14.3)	30 (71.4)
P20+PAM	6 (15.8)	6 (15.8)	26 (68.4)

Table S4 (Related to Figure 2D). Paired-Genotype Analysis of F2 Dumpy and Non-dumpy Animals from Single F1 Rollers.

Dpy Genotype	Non-Dpy Genotype	No-block (%)	PAM (%)	P2 (%)	P2+PAM (%)	P5 (%)	P8 (%)	P11 (%)	P14 (%)	P17 (%)	P20 (%)	P20+PAM (%)
HDR Edited	HDR Edited	0 (0.0)	3 (7.0)	3 (5.4)	3 (5.7)	2 (3.0)	1 (2.8)	4 (9.3)	1 (1.5)	1 (1.8)	5 (10.4)	4 (10.3)
	Indel	4 (4.8)	1 (2.3)	8 (14.3)	4 (7.5)	4 (6.1)	0 (0.0)	2 (4.7)	5 (7.6)	1 (1.8)	2 (4.2)	6 (15.4)
	Not Edited	16 (19.0)	33 (76.7)	32 (57.1)	40 (75.5)	42 (63.6)	22 (61.1)	28 (56.1)	34 (51.5)	19 (34.5)	24 (50.0)	24 (61.5)
Indel	HDR Edited	0 (0.0)	1 (2.3)	1 (1.8)	0 (0.0)	0 (0.0)	1 (2.8)	1 (2.3)	0 (0.0)	0 (0.0)	0 (0.0)	0 (0.0)
	Indel	1 (1.2)	4 (9.3)	0 (0.0)	0 (0.0)	0 (0.0)	1 (2.8)	0 (0.0)	1 (1.5)	4 (7.3)	2 (4.2)	0 (0.0)

	Not Edited	5	0	0	2	1	2	1	7	5	4	0
		(6.0)	(0.0)	(0.0)	(3.8)	(1.5)	(5.6)	(2.3)	(10.6)	(9.1)	(8.3)	(0.0)
	HDR	2	0	2	2	1	0	0	0	2	1	1
	Edited	(2.4)	(0.0)	(3.6)	(3.8)	(1.5)	(0.0)	(0.0)	(0.0)	(3.6)	(2.1)	(2.6)
Not Edited	Indel	4	0	3	0	1	1	0	1	2	2	1
		(4.8)	(0.0)	(5.4)	(0.0)	(1.5)	(2.8)	(0.0)	(1.5)	(3.6)	(4.2)	(2.6)
	Not Edited	52	1	7	2 (	15	8	7	17	21	8	3
		(61.9)	(2.3)	(12.5)	3.8)	(22.7)	(22.2)	(16.3)	(25.8)	(38.2)	(16.7)	(7.7)

Table S5 (Related to Figure 4A). HDR Incorporation Rates of Blocking Mutations and Non-blocking *Rsa*I Restriction Site Among HDR-edited Chromosomes

Blocking Mutation	*Rsa*I Only (%)	Blocking Only (%)	Blocking + *Rsa*I (%)
No-blocking	21 (100.0)	0 (0.0)	0 (0.0)
PAM	1 (2.2)	0 (0.0)	44 (97.8)
P2	15 (31.9)	15 (31.9)	17 (36.2)
P2+PAM	0 (0.0)	0 (0.0)	52 (100.0)
P5	1 (1.8)	40 (74.1)	13 (24.1)
P8	2 (8.0)	13 (56.0)	8 (36.0)
P11	0 (0.0)	21 (63.6)	12 (36.4)
P11+PAM	0 (0.0)	10 (27.8)	26 (72.2)
P14	8 (20.5)	22 (56.4)	9 (23.1)
P17	4 (15.4)	16 (61.6)	6 (23.0)
P20	4 (12.5)	17 (53.1)	11 (34.4)
P20+PAM	0 (0.0)	11 (28.2)	28 (71.8)

Table S6 (Related to Figure 3B). Effect of Distance to Cut Site on Incorporation of Single Nucleotide Guide Substitutions.

Blocking Mutation	PAM Only (%)	Blocking Only (%)	Blocking + *Rsa*I (%)
PAM	45 (100.0)	0 (0.0)	0 (0.0)
P2+PAM	0 (0.0)	0 (0.0)	52 (100.0)
P11+PAM	26 (72.2)	4 (11.2)	6 (16.6)
P20+PAM	28 (71.8)	0 (0.0)	11 (28.2)

Table S7 (Related to Figure 4). Blocking Efficacy of Single Nucleotide Substitutions

	Blocking Mutation	Both Reverted (%)	Heterozygous (%)	Not Reverted (%)
F1 Rol	No-blocking	0 (0.0)	25 (50.0)	25 (50.0)
	PAM	0 (0.0)	0 (0.0)	56 (100.0)
	P2	0 (0.0)	1 (1.8)	56 (97.2)
	P11	0 (0.0)	3 (5.4)	53 (94.6)
	P20	0 (0.0)	7 (12.7)	48 (87.3)
F1 Dpy	No-blocking	5 (10.0)	22 (44.0)	23 (46.0)
	PAM	0 (0.0)	0 (0.0)	50 (100.0)
	P2	0 (0.0)	3 (5.3)	53 (94.7)
	P11	0 (0.0)	3 (5.3)	53 (94.7)
	P20	1 (1.8)	12 (21.8)	42 (76.4)

Table S8 (Related to Figure 6). Editing Rates of *let-7* in F2 Generation non-Venus Animals.

Blocking Mutation	HDR Edited (%)	Indel (%)	Not Edited (%)
No-blocking	1 (3.7)	11 (40.7)	15 (55.6)
PAM	17 (60.7)	3 (10.7)	8 (28.6)
n2853 [P6]	17 (56.7)	7 (23.3)	6 (20.0)
